# The analysis of 2-amino-2-thiazoline-4-carboxylic acid in the plasma of smokers and non-smokers

**DOI:** 10.1080/15376510802488165

**Published:** 2009-06-30

**Authors:** Brian A. Logue, Wendy K. Maserek, Gary A. Rockwood, Michael W. Keebaugh, Steven I. Baskin

**Affiliations:** 1 Department of Chemistry and Biochemistry, South Dakota State University, Brookings, South Dakota, USA; 2 US Army Medical Research Institute of Chemical Defense, Aberdeen Proving Ground, Maryland, USA

**Keywords:** 2-amino-2-thiazoline-4-carboxylic acid, ATCA, Biomarker, Cyanide, Gas-chromatography mass-spectrometry

## Abstract

ATCA (2-amino-2-thiazoline-4-carboxylic acid) is a promising marker to assess cyanide exposure because of several advantages of ATCA analysis over direct determination of cyanide and alternative cyanide biomarkers (i.e. stability in biological matrices, consistent recovery, and relatively small endogenous concentrations). Concentrations of ATCA in the plasma of smoking and non-smoking human volunteers were analyzed using gas-chromatography mass-spectrometry to establish the feasibility of using ATCA as a marker for cyanide exposure. The levels of ATCA in plasma of smoking volunteers, 17.2 ng/ml, were found to be significantly (*p* < 0.001) higher than that of non-smoking volunteers, 11.8 ng/ml. Comparison of ATCA concentrations of smokers relative to non-smokers in both urine and plasma yielded relatively similar results. The concentration ratio of ATCA for smokers versus non-smokers in plasma and urine was compared to similar literature studies of cyanide and thiocyanate, and correlations are discussed. This study supports previous evidence that ATCA can be used to determine past cyanide exposure and indicates that further studies should be pursued to validate the use of ATCA as a marker of cyanide exposure.

## Introduction

Verification of exposure to cyanide by analysis of biological matrices is important in the analysis of both accidental exposure and deliberate release of cyanide. It is important for forensic applications (i.e. analysis that relates to establishing evidence and does not affect medical treatment) and diagnostic applications (i.e. analysis that establishes the presence of a medical condition). There are two major approaches used to verify cyanide exposure: (1) direct analysis of cyanide, and (2) analysis of the major cyanide metabolite, thiocyanate. Confirming cyanide exposure through direct analysis is difficult due to the consumption of cyanide in the daily diet, the volatility of hydrogen cyanide (HCN), the nucleophilic properties of cyanide ion (CN^−^), and the rapid metabolism of cyanide (half-life in plasma is reported to be from 4 minutes to approximately 1 hour; Hartung 1982; Lundquist et al. 1987; Baud et al. 1996; Baskin and Brewer 1997; Moariya and Hashimoto 2001).

Thiocyanate (SCN^−^) is the major product of cyanide metabolism. The formation of thiocyanate occurs when cyanide reacts with a sulfane sulfur donor (Isom and Baskin 1997), predominantly thiosulfate. This formation is enzymatically controlled by rhodanese (thiosulfate sulfurtransferase; Hebert 1993; Salkowski and Penney 1994; Tsuge et al. 2000; Baskin et al. 2004). Although it is the major product of cyanide metabolism, difficulties have been noted in the use of thiocyanate for verification of cyanide exposure. Thiocyanate is naturally found in biological fluids, and while this is a condition of all cyanide biomarkers, normal thiocyanate levels are very high and can be inconsistent (Diem and Letner 1970; Pettigrew and Fell 1973; Jarvis 1989; Maliszewski and Bass 1955; Yamanaka et al. 1991; Pre and Vassey 1992; Tsuge et al. 2000; Hasuike et al. 2004). Large and variable background thiocyanate concentrations (i.e. average SCN^−^ concentrations are approximately 33.1 μM, 60 μM, and 590 μM, for blood, urine, and saliva, respectively) make it difficult to determine low-level cyanide exposure from this metabolite. Ballantyne (1977) also found that concentrations of thiocyanate in blood varied unpredictably during storage at a number of temperatures and that analytical recovery of thiocyanate from whole blood was inconsistent. Large and variable concentrations may indicate that thiocyanate is involved in a number of biological processes in addition to cyanide metabolism. In fact, significant use of thiocyanate by biological processes other than cyanide metabolism has been established (Wood et al. 1947; Wood and Williams 1949; Isom and Baskin 1997).

**Figure 1:**
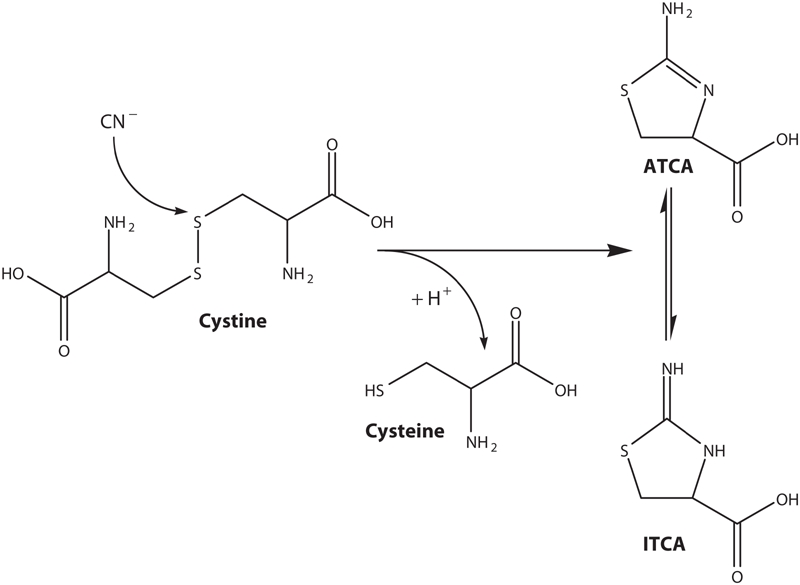
The production of ATCA from cyanide and cystine.

An alternative approach to verification of cyanide exposure is to analyze biological matricies for 2-amino-thiazoline-4-carboxylic acid (ATCA). The formation of ATCA from cyanide, as shown in Figure 1, appears to be an important biological pathway for cyanide detoxification (Salkowski and Penney 1994; Baskin and Brewer 1997; Borowitz et al. 2001). When cyanide reacts with cystine, ATCA or 2-iminothiazolidine-4-carboxylic acid (ITCA) is formed. These two tautomers, ATCA and ITCA, may be in equilibrium (Figure 1; Lundquist et al. 1995) and will be referred to as only ATCA. Approximately 15% of the initial dose of cyanide is converted to ATCA (Wood and Cooley 1956) by direct interaction of cyanide with cystine and not from other processes.

In biological samples, ATCA is stable for months at various temperatures (Lundquist et al. 1995; Logue et al. 2005). Relatively few techniques have been described to analyze ATCA from biological matrices, including spectrophotometry (Bradham et al. 1965; Baskin et al. 2006) and high-performance liquid chromatography (Lundquist et al. 1995). More recently, Logue et al. (2005) described a gas-chromatography mass-spectroscopic (GC-MS) method to analyze ATCA in human urine and reported concentrations of ATCA in smokers and non-smokers. Relatively few studies have been performed to evaluate the relationship between ATCA and cyanide, although recent studies have shed some light on this issue. Elevated concentrations of ATCA in the urine of smokers compared to non-smokers have been found (smokers inhale a small amount of cyanide while smoking; Lundquist et al. 1995; Harris 1996; Logue et al. 2005) and it has been established that ATCA is not metabolized further (Wood and Cooley 1956; Weuffen et al. 1980; Baskin et al. 2004). ATCA is excreted mainly in the urine (Baskin et al. 2004), and its toxicokinetics have not been specifically established. Although there are no specific toxicokinetic studies of ATCA, previous studies of the behavior of ATCA indicate that it has properties consistent with a lasting signature of cyanide exposure. Besides being a marker of cyanide exposure, ATCA may play a role in the neurotoxic effects of cyanide, as it appears to have some level of neurotoxicity (Bitner et al. 1997).

The relationship between cyanide exposure and markers of exposure must be understood in order to utilize a specific cyanide biomarker. If a strong relationship is established, forensic, clinical, military, scientific, and veterinary investigators could use this marker for diagnostic or forensic confirmation of cyanide exposure. The purpose of this study was to further evaluate the potential of ATCA for use as a valuable marker of cyanide exposure by evaluating the relationship between cyanide inhalation and ATCA concentrations in the plasma of smokers and non-smokers. These concentrations were compared to similar studies of ATCA, cyanide, and thiocyanate.

## Materials and methods

### Reagents

ATCA (full chemical name: L-2-amino-2-thiazoline-4-carboxylic acid) was obtained from Chem-Impex International (Wood Dale, IL). A standard stock solution (100 μg/mL) was prepared in 0.1 M HCl and used throughout the current study. Oasis solid-phase extraction (SPE) columns were obtained from Waters® Corporation (Milford, MA). The derivatizing agent N-methyl-N-trimethylsilyl-trifluoroacetamide (MSTFA) was purchased from Pierce Chemical Company® (Rockford, IL). The internal standard, deutereated ATCA (ATCA-d_2_), was obtained from the lab of Dr Herbert T. Nagasawa (Department of Veterans Affairs Medical Center, Minneapolis, MN), prepared by reaction of deuterated L-cysteine (3,3-d_2_) with cyanamide (Nagasawa et al. 2004). HPLC grade solvents and reagents were used throughout the study and were purchased from Sigma-Aldrich (St. Louis, MO).

### Biological fluids

Plasma samples from human subjects, age 18–60, were used to evaluate the applicability of the analytical method. Six male subjects (three smokers and three non-smokers) donated samples of blood at 8:00 a.m. (designated ‘morning’), at noon, and at 5:00 p.m. (designated ‘afternoon’) on three separate days over the course of a week (54 samples total). The smoking individuals (as defined by individuals who smoke at least five cigarettes per day with no other form of tobacco use) represent low level chronic exposure to cyanide through cyanide inhalation. Accurate intake of cyanide for individuals in this group is difficult to estimate because of variation in the number of cigarettes smoked, the cyanide dose per cigarette, the act of inhalation, etc. Therefore, these individuals will be referred to only as ‘smokers’.

Whole-blood samples were collected (2 mL) and mixed with EDTA. The samples were initially vortexed to ensure complete mixing prior to separation of the plasma and red blood cell fractions by centrifugation. The separate fractions were collected and stored in a freezer until analysis. To determine the effect of time variation on plasma concentrations of ATCA, smoking and non-smoking volunteers were separated into subgroups which were defined by days of week and time of day (e.g. smokers on Friday afternoon). Each subgroup was analyzed for significant difference (see ‘Calibration, quantification, and data analysis’ section). All samples were gathered under approved human use protocols from the gathering institution.

### Sample preparation

Samples were diluted 1:10 in 1 mL of 0.1 M HCl. Acidifying the plasma matrix ensured the amine moiety on ATCA (Figure 1) was protonated. Deuterated ATCA (100 μL of 1000 ng/mL ATCA-d_2_) was added to the diluted sample as an internal standard. After conditioning with successive washes of 1 mL each of methanol and H_2_O, the diluted sample was aspirated through an SPE column. The column was then washed successively with 1 mL each of 0.1 M HCl and 100% methanol. ATCA was then eluted into 2 mL micro-centrifuge tubes using 29% (w/w) NH_4_OH. Because highly basic solutions can chemically alter the ATCA structure, a small amount of concentrated HCl (200 μL) was added to the centrifuge tube to decrease the solution pH. The solvent was removed from the samples in a centrifugal evaporation system at 70°C for at least 6 hours. Derivatization of the dried samples was carried out at 50°C using 150 μL of 30% MSTFA in hexane for 1 hour in capped centrifuge tubes to produce trimethylsilyl-derivatized ATCA (ATCA-(TMS)_3_).

### Gas-chromatography mass-spectrometry

Analysis was performed on an Agilent GC-MS system consisting of a 6890 Series II gas chromatograph, a 5973N series mass-selective detector, and a 7683 auto-sampler. The column used was a DB-5MS bonded phase column (30 m × 0.25 mm I.D., 0.25 μm film thickness; J&W Scientific, Folsom, CA) with helium as the carrier gas at a flow rate of 0.1 mL/min. The GC oven temperature was initially held at 100°C for 1 minute, and then elevated at a rate of 15°C/min up to 250°C. The gradient was then increased at a rate of 30°C/min up to 280°C and held constant for 1 minute. The GC was interfaced with a mass-selective detector in selected ion monitoring (SIM) mode for monitoring abundant ions of ATCA-(TMS)_3_ (m/z 245, 347, and 362) and ATCA-d_2_ (m/z 349 and 364) with a dwell time of 100 ms each. Other parameters are reported by Logue et al. (2005).

### Calibration, quantification, and data analysis

Calibration samples were prepared in the range of 10–1000 ng/mL by diluting the stock ATCA solution in water as a surrogate and adding 100 ng/mL ATCA-d_2_ as an internal standard. Calibration was performed by plotting peak area ratios of ATCA to internal standard (ATCA-d_2_) of individual ions 362 and 364, respectively. The error involved in analyzing plasma samples for ATCA is reported in standard error of the mean throughout the text. Alternatively, it is sometimes useful to report the standard deviation of the data. For this study, the standard deviation for the analysis of ATCA in plasma was 5.2 ng/mL and 4.6 ng/mL for smokers and non-smokers, respectively. Comparison of the plasma samples for significant differences in the ATCA concentrations of smokers compared with non-smokers was accomplished using a two-tailed *t*-test. Comparison of each sub-group of smokers and non-smokers (e.g. non-smokers on Friday morning) was accomplished by a two-way analysis of variance and the Bonferroni multiple range test to identify significant differences between the identified subsets of volunteers.

## Results and discussion

### ATCA concentrations in smoker and non-smoker plasma

**Figure 2:**
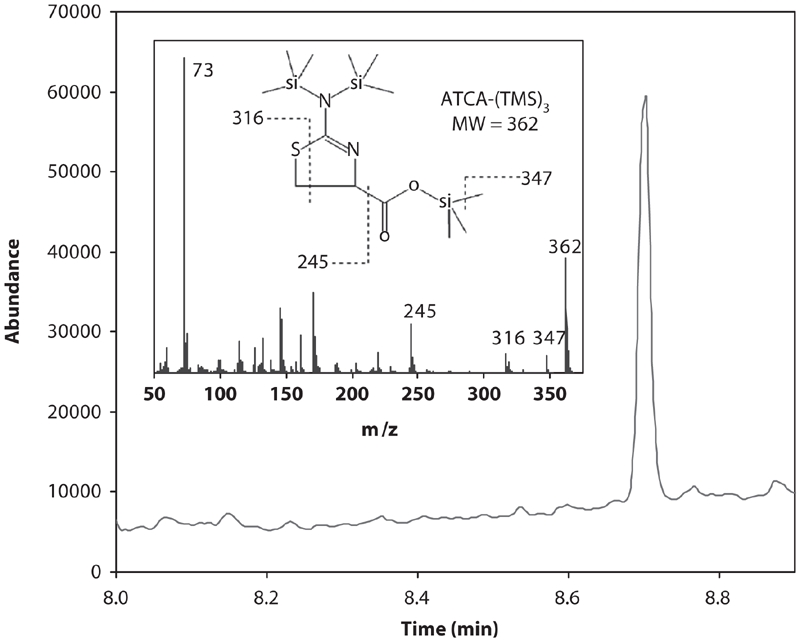
GCMS chromatograph of derivatized ATCA (ATCA-(TMS)_3_) in plasma. The chromatograph above is the total ion chromatograph (in selected ion mode; see ‘Materials and Methods’ for details) of the plasma of a smoking volunteer. The ATCA-(TMS)_3_ peak appears at 8.7 minutes. Inset is the full mass spectrum and chemical structure of ATCA-(TMS)_3_. Some of the ions in the mass spectrum are assigned above.

Human cyanide exposure can be evaluated directly through analysis of biological matrices of smokers or victims of accidental exposure (Chandra et al. 1980; Lundquist et al. 1987; Harris 1996; Their et al. 2000; Tsuge et al. 2000; Riddle 2004; Eckstein and Maniscalco 2006; Geller et al. 2006; Scherer 2006). Cyanide concentrations in the plasma, whole blood, saliva, and tissues of smokers are elevated with respect to non-smokers because a small amount of cyanide intake occurs during the act of smoking (Chandra et al. 1980; Lundquist et al. 1987; Harris 1996; Tsuge et al. 2000). There is also an inherent amount of cyanide present in blood samples and other biological tissue due to the consumption of various foods that contain cyanide and the breakdown of amino acids (Baskin and Brewer 1997; Ellenhorn and Barceloux 1997; Jones 1998). A portion of this inherent cyanide will presumably be converted to ATCA (Figure 1; Wood and Cooley 1956; Weuffen et al. 1980; Salkowski and Penney 1994; Harris 1996; Borowitz et al. 2001; Baskin et al. 2004). Therefore, as with cyanide and thiocyanate, there should be measurable endogenous levels of ATCA in human biological samples. In fact, endogenous concentrations of ATCA were found in human urine samples, with smokers having elevated concentrations relative to non-smokers (Logue et al. 2005). Therefore, ATCA concentrations for smokers should also be elevated above background levels of ATCA found in non-smoker plasma. A representative chromatograph for the analysis of ATCA from the plasma of a smoker is presented in Figure 2. As with all cyanide markers, significant endogenous concentrations of ATCA dictates that ‘normal’ background levels of ATCA for both smokers and non-smokers must be known for each individual biological matrix. Background levels must be known so that significantly elevated levels of ATCA, which may indicate cyanide exposure, can be determined.

**Figure 3:**
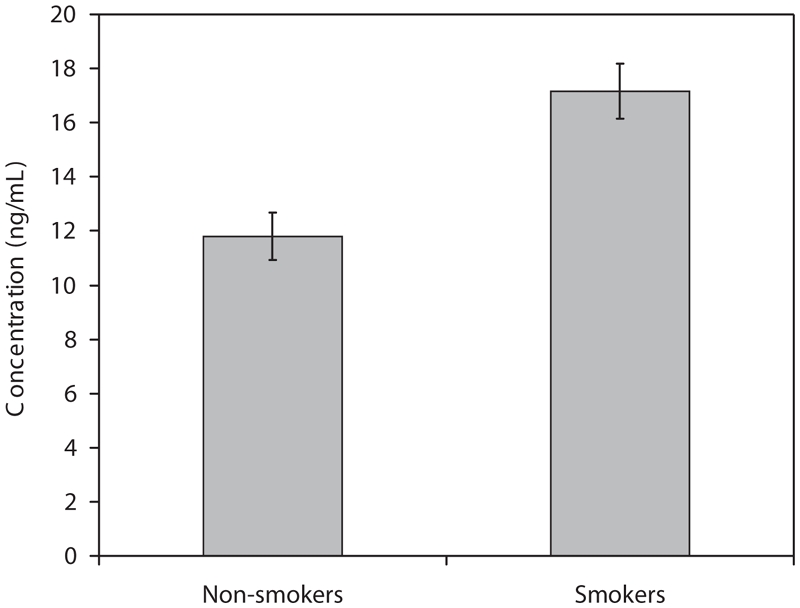
ATCA concentrations in the plasma of smokers compared to non-smokers (error bars indicate the standard error of the mean for each group). The concentration of ATCA was found to be significantly elevated in the plasma of smokers (p < 0.001).

The concentration of ATCA in the plasma of smoking and non-smoking volunteers was evaluated to determine whether there was a significant increase in ATCA concentrations of smokers relative to non-smokers and to estimate background levels of ATCA in the plasma of smokers and non-smokers. The plasma concentration of ATCA in the volunteers was 17.2 ± 1.0 ng/mL and 11.8 ± 0.88 ng/mL for smokers and non-smokers, respectively (Figure 3). Comparing these two averages with a two-tailed *t*-test indicates that a significant difference exists between these two groups at a 99.9% (*p* < 0.001) confidence level.

### Evaluation of time dependence of ATCA exposure

Plasma samples were gathered throughout each day and over the course of a 1-week period. Overall, the ATCA concentration ranged from 7.03–14.6 ng/mL for non-smokers and 13.6–19.4 ng/mL for smokers throughout the various times during the week (Table 1). Each sub-group (e.g. non-smokers at Wednesday noon) was analyzed for significant difference. Although the amount of cyanide intake for smokers and non-smokers can vary throughout the day and the week, the concentration of ATCA did not significantly change (ANOVA analysis *p*-values ranged from 0.84–0.35; the Bonferroni test also confirmed that there were no significant differences between these groups). The lack of significant variation of ATCA concentrations between the day of the week and the time of day indicates that the ATCA concentrations are fairly stable over time. This would not be expected if the distribution and depuration half-lives were short (i.e. 1–2 hours); the ATCA concentration would increase throughout the day. If the distribution and depuration half-life of ATCA are long (i.e. multiple hours), fairly stable concentrations of ATCA would be seen.

**Table 1:** ATCA concentrations in the plasma of smokers and non-smokers during various times during a 1-week period. The error for each entry is reported in standard error of the mean (*n* = 3 for each group).

	Monday		Wednesday		Friday	
			
	Smokers (ng/mL)	Non-smokers (ng/mL)	Smokers (ng/mL)	Non-smokers (ng/mL)	Smokers (ng/mL)	Non-smokers (ng/mL)
Morning	19.2 ± 0.9	12.5 ± 4.0	14.7 ± 3.5	14.6 ± 1.3	18.7 ± 2.4	14.2 ± 2.0
Noon	13.6 ± 3.1	7.03 ± 1.6	15.3 ± 2.9	13.1 ± 2.2	18.7 ± 2.3	11.7 ± 1.4
Afternoon	18.1 ± 2.4	11.0 ± 3.5	19.4 ± 6.7	9.96 ± 2.6	16.7 ± 2.4	12.4 ± 4.4

The concentrations of ATCA for each sub-group in Table 1 are consistently larger for smokers than for non-smokers. Although all ATCA concentrations are larger for smokers, the difference between smokers and non-smokers on Wednesday morning is insignificant. This is most likely due to the limited number of samples gathered for each sub-group (*n* = 3) and the error involved in analyzing human plasma samples for ATCA. This sub-group illustrates that, although tentative conclusions can be made from these sub-groups, a more extensive study should be completed to validate the data.

### Comparison of ATCA concentrations in human biological matrices

Direct comparison of urine and plasma is not possible, but ATCA concentrations for smokers in both urine (Logue et al. 2005) and plasma (this study) matrices are significantly higher relative to non-smokers (saliva concentrations of ATCA have thus far not been analyzed for smokers and non-smokers). ATCA concentrations in urine were 115 ± 13 ng/mL and 87 ± 12 ng/mL for male smokers and non-smokers, respectively (standard error of the mean given; Logue et al. (2005) reported standard deviations of 46 and 36 ng/mL for male smokers and non-smokers, respectively) while plasma concentrations were 17.2 ± 1.0 ng/mL and 11.8 ± 0.88 ng/mL for smokers and non-smokers.

**Figure 4:**
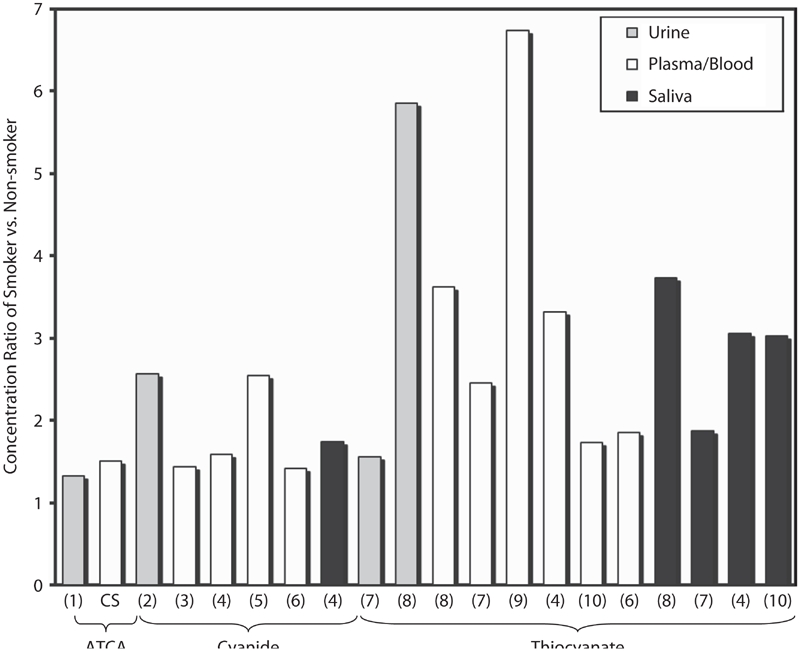
The concentration ratio (smokers/non-smokers) of cyanide biomarkers (cyanide itself, SCN−, and ATCA) in the urine, blood, or saliva of human smokers and non-smokers. Similar studies were differentiated by the biological matrix (blood, urine, saliva) and the analyte studied (ATCA, cyanide or thiocyanate), as indicated in the figure. The number below each bar indicates the reference from which the ratio was calculated: CS indicates ‘current study’; 1. Logue et al. (2005); 2. Sano et al. (1989); 3. Lundquist et al. (1987); 4. Tsuge et al. (2000); 5. Lundquist et al. (1985); 6. Hasuike et al. (2004); 7. Jarvis (1989); 8. Maliszewski and Bass (1955); 9. Pre and Vassey (1992); 10. Yamanaka et al. (1991). The ratios of cyanide biomarkers were 1.32–1.51 for ATCA, 1.44–2.56 for cyanide, and 1.56–6.74 for thiocyanate.

While comparison of absolute concentrations of cyanide biomarkers in different biological matrices is not possible, comparison of the ratio of smokers relative to non-smokers is possible (e.g. SCN^−^ concentration in smokers' plasma/SCN^−^ concentration in non-smokers' plasma). The ratios of mean ATCA concentrations of smoker to non-smoker were analyzed from both urine and plasma matrices (Logue et al. 2005; current study; Figure 4). These ratios were also compared to cyanide and thiocyanate ratios from similar studies (Maliszewski and Bass 1955; Lundquist et al. 1985, 1987; Jarvis 1989; Sano et al. 1989; Yamanaka et al. 1991; Pre and Vassey 1992; Tsuge et al. 2000; Hasuike et al. 2004) in a number of matrices (blood/plasma, saliva, and urine; presented in Figure 4). The ratios are strikingly similar for ATCA between plasma and urine with a calculated ratio of 1.51 and 1.32, respectively. The ATCA ratio is also similar to the cyanide ratio, which ranged from 1.44–2.56 for other similar studies (Lundquist et al. 1985, 1987; Sano et al. 1989; Tsuge et al. 2000; Hasuike et al. 2004). The similarity of the ratios between cyanide and ATCA provides evidence that ATCA production may be exclusively, or at least predominantly, from cyanide metabolism, and that there may be a direct link between elevated ATCA concentrations and cyanide exposure. Although cyanide ratios show some inconsistency (Figure 4), the range of these ratios is relatively small considering the difficulties in cyanide analysis and that different analytical methods were used to analyze cyanide in these studies. As opposed to ATCA and cyanide ratios, the ratios of thiocyanate are very inconsistent between studies (Maliszewski and Bass 1955; Jarvis 1989; Yamanaka et al. 1991; Pre and Vassey 1992; Tsuge et al. 2000; Hasuike et al. 2004) and even within the same study for different biological matrices (Jarvis 1989; Yamanaka et al. 1991). The thiocyanate ratio ranged from 1.56–6.74. The ratio of thiocyanate for most of the studies in Figure 4 is larger than the ratio of ATCA or cyanide, which is an advantage for analyzing thiocyanate. The inconsistency of the thiocyanate ratio may indicate that production or depletion of thiocyanate by sources other than cyanide metabolism may be occurring. The variability of the thiocyanate ratio may also be caused by fast kinetics of thiocyanate production and depletion, where biological matrices gathered at different times may have quite different thiocyanate concentrations depending on the biological process most relevant. Another explanation as to the variability of thiocyanate between biological matrices is the highly variable tissue distribution of rhodanese (Himwich and Saunders 1948; Baskin et al. 2004).

Figure 4 indicates that thiocyanate may not be a good marker of cyanide exposure because of highly variable endogenous concentrations. Scherer (2006) has also suggested that thiocyanate is limited as a marker of cyanide exposure. Comparing the ratios of ATCA and thiocyanate to cyanide indicates that ATCA may be a better-suited marker for determining cyanide exposure. It should be noted that some similar studies were excluded from Figure 4 because the high variability in the data was such that the difference between smokers and non-smokers of the species analyzed was insignificant. Consideration of excluded studies still supported the conclusions reached from this treatment of the data.

These results, along with previous studies evaluating ATCA as a possible marker of cyanide exposure, continue to provide evidence that ATCA can be utilized as a marker for cyanide poisoning in forensic and possibly diagnostic applications. The current study may be useful in identifying non-smoking individuals who may be chronically overexposed to cyanide in occupations where cyanide is extensively utilized (e.g. gold mining operations). Future work must evaluate the behavior (i.e. formation, distribution, and excretion) of ATCA during and after controlled cyanide exposures. A relationship should also be established between the dose of cyanide and the formation of ATCA. Overall, this study further supports the use of ATCA as a marker of cyanide poisoning.
